# Supplementary material for: Growth Factor Screening in Dystrophic Muscles Reveals PDGFB/PDGFRB-Mediated Migration of Interstitial Stem Cells
Source: Int J Mol Sci. 2019 Mar 5;20(5):1118. doi: 10.3390/ijms20051118 (PMC6429448; doi:10.3390/ijms20051118)
Supplement: Supplementary file 1 [file ijms-20-01118-s001.zip › supplementary files/ijms-448273-supplementary.pdf]

A

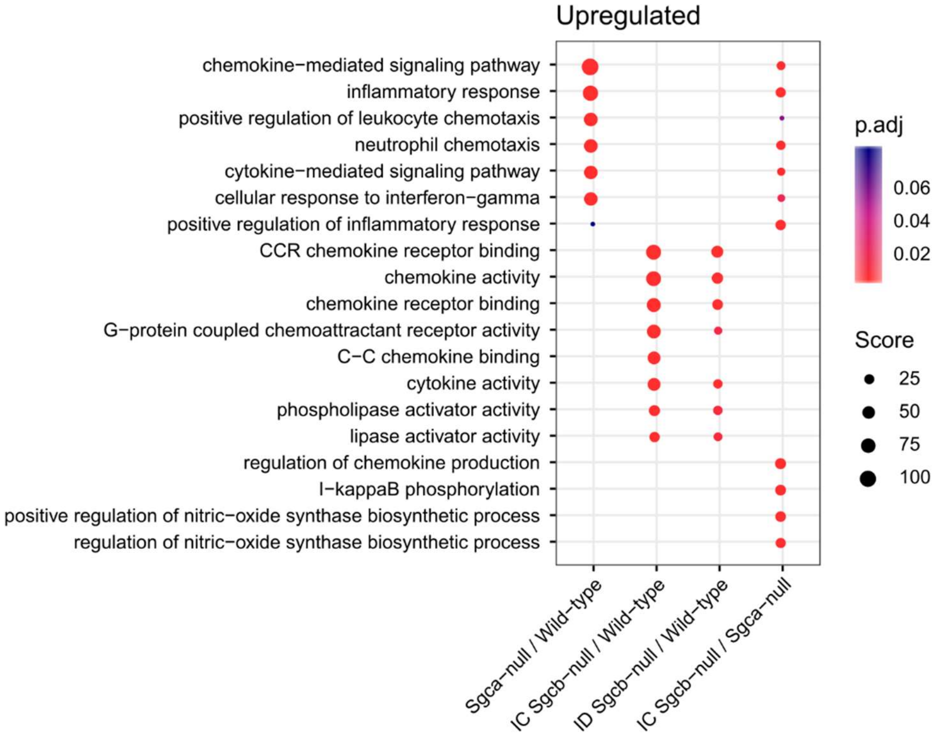

B

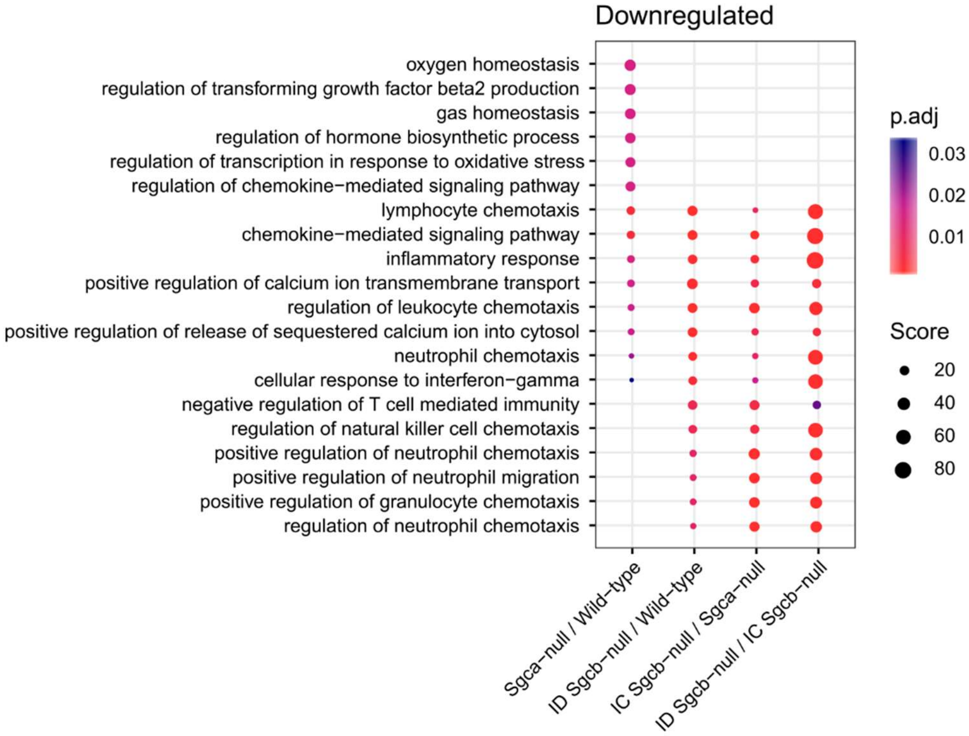

Supplementary Figure 1: Gene set enrichment of differentially expressed chemokines between dystrophic hearts. GO analysis on biological processes of differentially upregulated genes (**A**) and differentially downregulated genes (**B**) between Wild-type, *Sgca*-null, IC *Sgcb*-null and ID *Sgcb*-null hearts. Top 6 scoring enrichment terms per group selected.
