# Supplementary figures and images for: Growth Factor Screening in Dystrophic Muscles Reveals PDGFB/PDGFRB-Mediated Migration of Interstitial Stem Cells
Source: Int J Mol Sci. 2019 Mar 5;20(5):1118. doi: 10.3390/ijms20051118 (PMC6429448; doi:10.3390/ijms20051118)

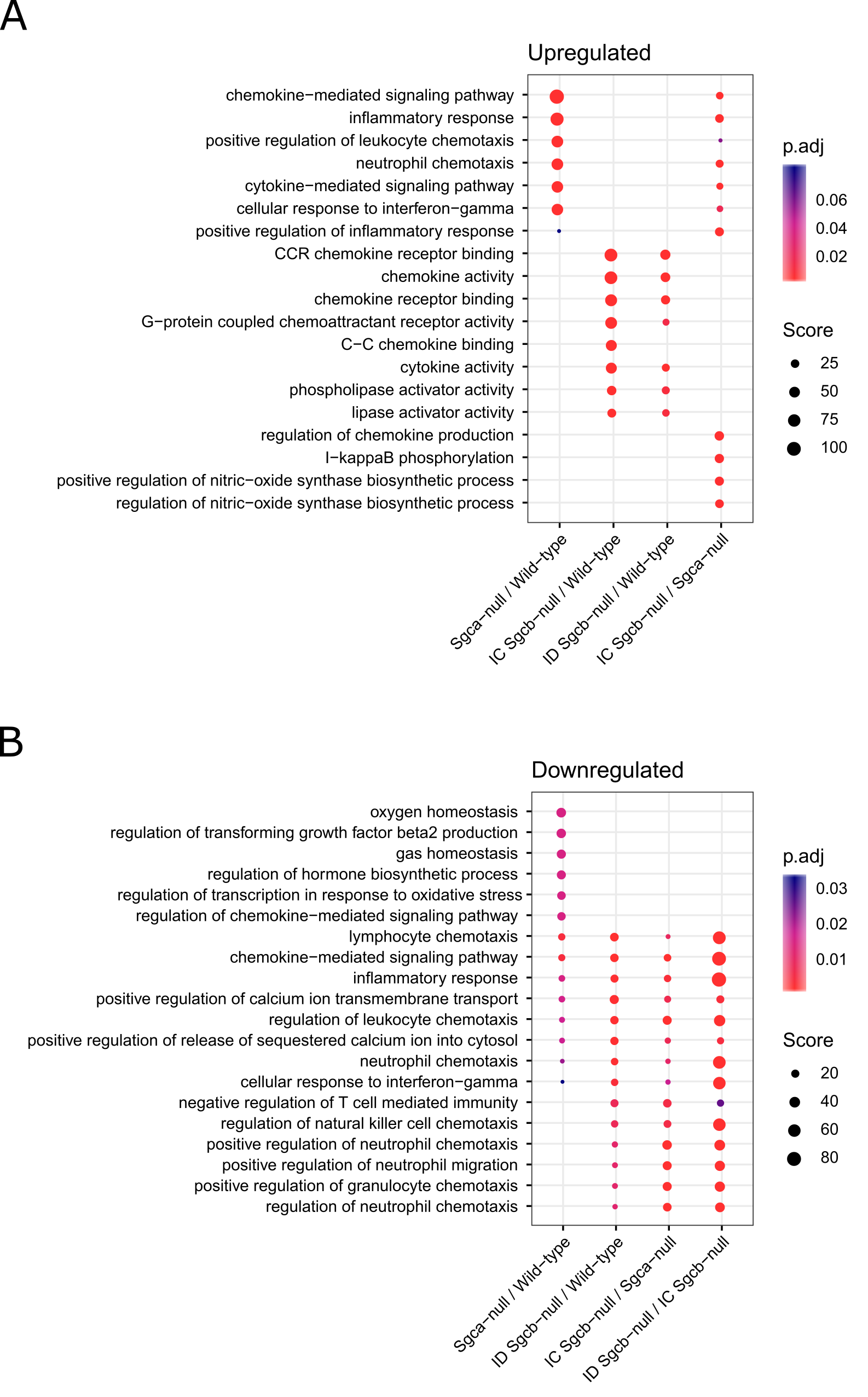

Supplement: Supplementary file 1 [file ijms-20-01118-s001.zip › supplementary files/Suppl Fig1 GO analysis.png]
